# Level of Sedentary Behavior and Its Associated Factors among Saudi Women Working in Office-Based Jobs in Saudi Arabia

**DOI:** 10.3390/ijerph14060659

**Published:** 2017-06-19

**Authors:** Nada M. Albawardi, Hoda Jradi, Abdulla A. Almalki, Hazzaa M. Al-Hazzaa

**Affiliations:** 1 King Abdullah International Medical Research Center, Community and Environmental Health, College of Public Health and Health Informatics, King Saud Bin Abdulaziz University for Health Sciences, Riyadh 11426, Saudi Arabia; jradih@ksau-hs.edu.sa; 2 Health Sciences Research Center, Princess Nourah Bint Abdulrahman University, Riyadh 11671, Saudi Arabia; ab.almalki@hotmail.com; 3 Emeritus Professor and Former Director of Pediatric Exercise Physiology Research Laboratory, King Saud University, Riyadh 11673, Saudi Arabia; halhazzaa@hotmail.com or alhazzaa@ksu.edu.sa

**Keywords:** physical activity, sedentary behavior, obesity, overweight

## Abstract

Research in Saudi Arabia has revealed a shocking level of insufficiently physically active adults, particularly women. The risk of sedentary behavior will likely increase as the number of women with office-based jobs increases. The aim of this study is to determine the level of sedentary behavior, and its associated factors, among Saudi women working office-based jobs in the city of Riyadh. A cross-sectional study of 420 Saudi female employees at 8 office-based worksites were measured to determine body mass index and were given a self-administered survey to evaluate their level of physical activity and sedentary behavior. Median sitting time on work days was 690 min per day (interquartile range, IQR 541–870), with nearly half accumulated during work hours, and 575 min per day (IQR 360–780) on non-work days. Predictors of work day sitting time were level of education, number of children, and working in the private sector. Number of children, whether they were single, and whether they lived in a small home were found to predict non-work day sitting time. This study identifies Saudi women in office-based jobs as a high-risk group for sedentary behavior. There is a need to promote physical activity at worksites and reduce prolonged sitting.

## 1. Introduction

Rapid development of modern technology, transportation, and communications has not only decreased the demand for physical activity (PA), but has also caused sitting to become one of the most common postures in today’s workplace, recreational activities, and daily commuting. Research on sedentary behaviors, defined as “those that involve sitting and low levels of energy expenditure (1–1.5 METs (metabolic equivalent of task))” [1], has proliferated over the past decade; objective assessments of sedentary behavior revealed positive associations with cardio-metabolic biomarkers [2] and increased all-cause and cardiovascular disease (CVD) mortality [3]. Moreover, sedentary behaviors were shown to be associated with negative health outcomes differing from those attributed to physical inactivity [4].

Research on physical inactivity prevalence in the Kingdom of Saudi Arabia (KSA) has increased in the past decade, exposing a shocking level of insufficiently physically active adults, especially women [5,6]. In fact, the World Health Organization (WHO) reported in 2010 that 74.9% of Saudi women were insufficiently active, making them among the least physically active worldwide [7]. While studies have explored the relationship between occupational status and PA [8,9], no studies have examined the PA levels or sedentary behaviors of working adults in this region. As the number of women entering the KSA workforce grows [10,11], office-based jobs may increase the risk of sedentary behavior for this already vulnerable group. The aim of this study was to determine the level of sedentary behavior and its associated factors among Saudi women employed in office-based jobs in Riyadh.

## 2. Materials and Methods

### 2.1. Design and Setting

Purposeful sampling of worksites in Riyadh, Saudi Arabia, was used in the cross-sectional design. The inclusion criteria included jobs that are largely office-based (necessitating little physical work) and work areas that are segregated by gender (commonly practiced in the KSA). To increase generalizability, worksites of varying sizes and identified as public, private, or philanthropic organizations were included. A ratio of 2:1 public to private worksites were chosen to attain employees similar to the ratio of females employed in the general public [10,11]. Eleven organizations were approached, including government offices, private companies, academic institutions, financial institutions, and charitable organizations. Eight organizations agreed to take part in this study.

### 2.2. Study Sample

To be eligible, participants were required to be female Saudi nationals between the age of 18 and 60 years and working in a primarily office-based setting. As this study was a census, the researcher or a staff member who was informed of the goals and significance of the study recruited all available employees who met the inclusion criteria. Of the 586 eligible employees approached, 420 agreed to participate, resulting in a response rate of 72%.

### 2.3. Study Instruments

A self-administered survey was distributed using hard copies, which included demographic information, psychosocial constructs, and questions on physical activity and sedentary behavior.

#### 2.3.1. Sedentary Behavior

Sedentary behavior was evaluated by calculating sitting time using five questions from the Workforce Sitting Questionnaire (WSQ), which included reporting the number of minutes per day the participants spent sitting while working, commuting, using a home computer, watching television, and during recreation on work days and non-work days [12]. The WSQ test–retest reliability was previously evaluated at fair to excellent (ICC = 0.46–0.90) and had sufficient criterion validity against accelerometry in women (*r* = 0.22–0.46) [12]. Total sitting time on work days and non-work days was calculated using the sum of minutes spent sitting per day for all activities. The WSQ was forward and back translated into the Arabic and English languages, respectively.

The frequency that the subjects engaged in prolonged sitting (over 30 min) was also assessed using a five-point scale. In addition, the subjects’ beliefs on whether sedentary behavior could negatively impact health were assessed by two items using a five-point Likert scale. Subjects were asked if they agreed that “sitting for prolonged periods can negatively impact my health” and that “sitting for prolonged periods will not negatively impact my health if I regularly exercise after work”.

#### 2.3.2. Physical Activity

Physical activity was assessed using questions adapted from the Arab Teens Lifestyle Study Questionnaire, which was shown to have an acceptable validity coefficient against an electronic pedometer (*r* = 0.37; *p* < 0.001) [13]. Subjects were asked to report how many minutes (on how many days per week), they usually engage in various PAs, such as housework, walking for exercise, walking as a means of transport, taking the stairs in the workplace and outside of work, and moderate and vigorous activities. PA level was classified by calculating total “metabolic equivalents” of all activities per minute per week (MET min/week). The PA compendium [14] was then used to classify subjects as having low (<600 MET min/week), moderate (600–2999 MET min/week), or high activity (≥1500 MET min/week vigorous activity or ≥3000 MET min/week moderate/vigorous PA).

#### 2.3.3. Psychosocial Constructs

General self-efficacy and social support for PA were also assessed, as these two psychosocial constructs are recognized as being significantly associated with PA. The General Self-Efficacy Scale [14] has been extensively tested for evaluating self-efficacy, yielding internal consistency alpha values ranging between 0.75 and 0.91 for 25 countries [15]. It is comprised of ten items scored using a four-point Likert scale. In this study, the general self-efficacy score was calculated from the mean of the responses to the 10 items. The Physical Activity Social Support (PASS) survey [16] has been shown to have adequate validity and reliability in evaluating social support [16]. The possible mean scores of the five items in this survey range from −18 to 30. To assist in the interpretation of results, both the General Self-Efficacy Scale and PASS survey were categorized as “high” or “low” using the median scores of the respondents. Translation into Arabic and back translation into English were conducted for both the PASS survey and the General Self-Efficacy Scale. Fifteen subjects then completed the surveys in both languages, yielding 93.8% agreement between responses. The complete survey was validated for comprehension, clarity, feasibility, and test–retest reliability by 21 subjects similar to the study sample.

#### 2.3.4. Body Mass Index

To determine the participants’ body mass index (BMI) scores (kg/m^2^), measurements of height and weight were taken using a Seca 213 portable measuring rod (Seca, Hamburg, Germany) and Seca 813 digital floor scale. Using the BMI scores, subjects were then categorized as underweight (<18.50), normal weight (18.50–24.99), overweight (25.00–29.99), or obese (≥30.00) according to the international classification system used by the WHO. Pregnant subjects (*n* = 8) were not measured.

### 2.4. Ethical Issues

Ethical approval was obtained from the Institutional Review Board at King Abdullah International Medical Research Center (RR13/002) under the reference RRSS/005/2013. The study was approved to collect data from all work areas, and informed consent was obtained from all participants.

### 2.5. Data Analysis

SPSS version 20 (Armonk, NY, USA) was used to perform the statistical analyses. Total sitting time was capped at 960 min per day (as this would allow for eight hours of sleep) and was represented by the median and interquartile range (IQR). Continuous demographic and psychosocial variables were reported as mean and standard deviation, and categorical variables as frequency and valid percent. General Self-Efficacy Scale and PASS survey were categorized as “high” or “low” using the median scores of the respondents.

Mean and median sitting times during the individual activities of commuting, using a computer, watching TV, or socializing on work days and non-work days were compared and reported. Responses on how frequently respondents sat for periods of over 30 min during work hours were described using frequency and valid percent, and significant associations with demographic variables were reported. The frequencies and valid percentages of the respondents’ beliefs about the effect of sedentary behavior on health were also recorded.

Differences between participants in demographic and psychosocial characteristics, and mean sitting time on work days and non-work days, were evaluated using the Kruskal–Wallis test, where a *p*-value less than 0.05 was considered significant. Predictors for sitting time were determined using multiple linear regression analyses and stepwise elimination of variables, and significance was declared at *p* < 0.05 and 95% confidence interval (CI).

## 3. Results

The sample demographic characteristics are displayed in Table 1. The mean age of the respondents was 31.7 (±8.3). The greatest proportion of respondents (38.2%) were in the “normal” BMI category; however, over half of the sample were either “overweight” or “obese” (58.3%). The mean BMI was 27.1 (±5.9), which lies in the “overweight” category. The majority of the sample reported “low” PA levels (52.1%).

Median sitting times during the individual activities of commuting, using a computer, watching TV, or socializing were significantly greater on non-work days than on work days (Table 2). However, due to the reports of higher sitting time at work (343.9 min/day (240–420)), total sitting time was significantly greater on work days than on non-work days (*p* < 0.001).

**Figure 1:**
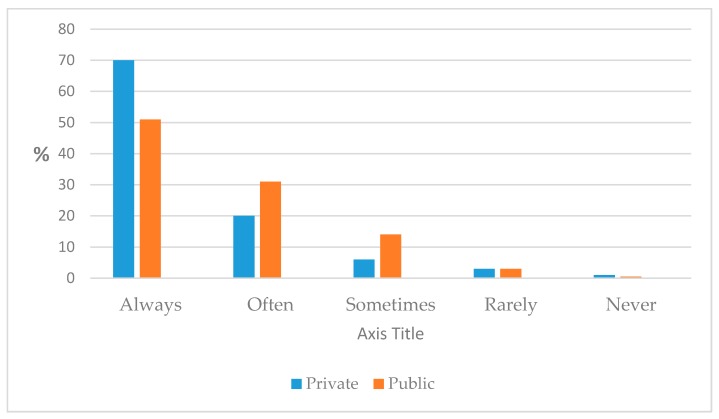
Proportion of public-and private-sector workers sitting for periods of over 30 min during work.

More importantly, 84.7% of all respondents reported that they often or always sit for periods of 30 min or more during work. This was significantly more common among private-sector workers, compared to public-sector workers (*p* = 0.004) (Figure 1).

The two items exploring the respondents’ beliefs about the effect of sedentary behavior on health revealed that while the majority (95.2%) agreed that prolonged sitting can negatively impact health, 49.1% falsely believed that exercising after work (after prolonged periods of sitting) would offset the negative impact of prolonged sitting (Table 3). Whether the subjects responded that they “agreed/strongly agreed” or “disagreed/strongly disagreed” that prolonged sitting would negatively impact their health if they exercise after work (item 2) was not significantly associated with sitting times on work (*p* = 0.125) and non-work days (*p* = 0.812).

Table 4 displays the median (IQR) sitting time on work days and non-work days among various demographics. General Self-Efficacy Scale and PASS survey were categorized as “high” or “low” using the median scores of the respondents (3 and 11, respectively). These variables were used to predict sitting time using multiple linear regression analyses and stepwise elimination, resulting in the model presented in Table 5.

The significant predictors for sitting time on work days included number of children, job sector, and education level (Table 5). Sitting time was projected to decrease by approximately 37 min with each additional child. Private-sector workers were predicted to increase sitting time by nearly 80 min per day on work days, compared to public-sector and philanthropic employees. Work day sitting was also predicted to increase by 55 min as education level increased. This model explained 21% of the variance in sitting time on work days. The model for non-work day sitting shows that the number of children, the size of home, and marital status are significant factors. Sitting on non-work days was projected to decrease by 27 min with each additional child. Living in a small home, as compared to a medium home, reduced sitting time by 81 min per day. Being single, as compared to married, increased sitting time by 73 min per day. However, this model only explained 10% of the variance in sitting time on non-work days.

## 4. Discussion

Sedentary behavior is a probable independent predictor for certain health outcomes, as it is shown to be associated with different negative health outcomes from those attributed to physical inactivity [4], including cardio-metabolic biomarkers [2] and increased all-cause and CVD mortality [3]. Thresholds for adverse health outcomes have not been established due to a lack of data and variations in reporting. Sitting time was self-reported in this study, as it has been found to be an acceptable tool for determining health-outcome associations in cross-sectional studies [16]; however, these reports have been shown to consistently underestimate sitting time [17]. To mitigate this issue, this study measured sitting time during domain-specific activities, such as sitting during transport and sitting at work, that have been found to have a greater correlation with objectively tested sitting time. Additionally, sitting times during the domain-specific activities were shown to be less prone to underreporting, compared to single-item sitting time for the entire day [16]. 

The findings show that the total sitting time on work days have a median of 690 min per day (IQR 541–870), with nearly half accumulated during work hours. Respondents reported nearly 20% less sitting time on non-work days. This is similar to figures in the International Prevalence Study [17] of adults aged 18–65 years in 20 countries, in which Saudi Arabia reported the highest proportion of its sample could be found in the 5th quintile of 540–1020 min, while the median of the entire international sample was 300 min per day (IQR 180–480). 

Jans et al. [18] reported less sedentary time among Dutch workers (*n* = 7720), compared to the present study, with an average of seven hours of sitting time, a third of which was accumulated at work. A sample of working adults from Australia (*n* = 1048) reported substantially lower sedentary behavior than those in the present study, with sitting times of 348 min per day on work days and 264 min per day on non-work days [19].

The lower average figures from these international studies might be explained by their use of data from representative samples [17] or working adults in general [18,19]. The present study assessed office-based workers only, which are more likely to report greater sitting times at work; however, even non-work day sitting times were much greater among the present sample than those reported in other studies. 

In addition to the total sitting time, the high frequency of prolonged sitting (85%) reported by this sample poses an additional health risk (independent of total sedentary time and physical inactivity) [20,21]. Thorp et al. [22] conducted an objective study on sitting times among office, call center, and service desk personnel, finding that approximately half of these times were over a period of 20 min or more. As beneficial health effects (such as significantly lower waist circumference, BMI, triglycerides, and two-hour plasma glucose) have been associated with interrupting long periods of sitting [20,23], accurate assessments of this behavior using objective measures are warranted, as this association may have important public health implications.

Correlates of sedentary behavior have not been as extensively reported as those for physical activity. Identifying demographics who may be at higher risk for sedentary behavior will allow for more focused PA programs in the workplace. In this sample, the only predictor for significantly higher sitting times on both work and non-work days was the number of children. Sitting time was projected to decrease by approximately 37 min per day on work days and 27 min per day on non-work days with each additional child. This variable has not been extensively studied in existing literature [24,25]. O’Donoghue et al. [25] systematically reviewed 74 studies on correlates of sedentary behavior, yet only 8 studies reported on the presence of children in the household. Five of these studies found lower overall sitting times among people who have children [26,27,28,29,30]; however, two reported higher sitting times while commuting [27,31]. Busschaert et al. [32] found that adults with children sat 21% less while using a home computer. This may reflect the important role that having children and increased physical responsibilities plays in decreasing sedentary behaviors, while also identifying women without children as a priority target for campaigns to reduce sitting time.

Among the present sample, education level was a significant predictor for sedentary behavior on work days. Sitting time on work days is predicted to increase by 55 min per day as education level increases. In previous studies, the relationship between education and sedentary behavior depended on the domain of sedentary time measured; screen time had an inverse relationship with education level, whereas total sedentary time was positively correlated [25]. It appears that professional occupations for women with higher education levels require higher sitting times, which increases total sedentary time.

It is interesting that work sector was also a predictor for sedentary behavior on work days, with private-sector workers projected to sit 80 min more per day than those working in other sectors. Private-sector workers were also significantly more likely to report sitting for periods of 30 min or more during work. No studies were found that evaluated this factor and its correlation with sedentary behavior. Whether this reflects a more stringent and competitive work culture within the private sector of the KSA is not clear and warrants further investigation.

Single women in this sample were found to be at greater risk for sedentary behavior on non-work days. Previous reports in eight studies investigating the association between marital status and sedentary behavior are inconsistent [24,25], showing increased, decreased, or no association with marital status.

In this sample, living in a small house was a significant predictor for increased sitting on non-work days, as compared to living in a medium-sized home. Only one study was found that considered the type of the home, showing objectively measured levels of sedentary behavior among those living in an apartment or a duplex were 2% higher than among those living in a house [33]. Presence of a domestic helper or other family member that may have an effect on the subject’s responsibilities at home was not explored in this study and may play a mitigating factor on the amount of PA and sitting time and the type (size) of home.

This study had several limitations. First, the main instrument for data collection was a survey, which is subject to social desirability and recall bias. While the survey was reliable and valid, it is recommended that future studies consider more objective instruments for assessing sedentary behavior, such as accelerometers. Second, the ability to make causal inferences was limited by the cross-sectional design. As the sample was from Riyadh, the generalizability of the findings to other regions of the KSA is limited. Third, this study explored several individual and psychosocial correlates to sedentary behavior but did not include environmental factors at home, at work, and in the community, which other studies have shown to be of importance. Finally, a lack of comparable data in the region, especially from this subset of the population, also limited the extent of analysis in the present study.

## 5. Conclusions

The magnitude of sedentary behavior among this sample of Saudi women was generally greater than what had previously been reported among other populations. Societal restrictions on the types of occupation currently available to Saudi women may pose unique risks for those with office-based jobs. With a growing number of Saudi women entering the workplace, studying the effect of workplace sedentary behavior on health is warranted. This may also support the use of workplace wellness programs to reduce sitting time and promote physical activity, thereby reducing chronic diseases and their risk factors as part of a holistic public health approach. Identifying correlates of sedentary behavior within this population will allow for the development of more effective programs that prevent the negative consequences of sedentary behavior. This study also identified segments of the population that may require greater attention when planning public health initiatives to decrease sedentary behavior, including women without children, those with higher education levels, and those working in the private sector. This study focused on the effect of individual and social factors on sedentary behavior, and further exploration of environmental factors, particularly those in the built environment, is warranted. Future research on the magnitude of sedentary behavior among women in other occupations and in other regions of the KSA is also recommended.

## Figures and Tables

**Table 1:** Demographic variables among the sample of Saudi female employees (*n* = 420).

Variables	*n* (Valid Percent)
Age Range (*n* = 317)	
Mean (SD): 31.7 (±8.3)	
18–25	67 (21.1)
26–35	176 (55.5)
36–45	45 (14.2)
46–60	29 (9.1)
Marital Status (*n* = 415)	
Single	97 (23.4)
Married	185 (44.6)
Divorced	106 (25.5)
Widowed	27 (6.5)
Number of Children (*n* = 416)	
0	200 (48.1)
1	50 (12.0)
2	44 (10.6)
3	43 (10.3)
4	40 (9.6)
5 or more	39 (9.4)
Education Level (*n* = 416)	
Primary school or lower	5 (1.2)
Middle school	7 (1.7)
High school	53 (12.7)
College diploma/bachelor’s degree	334 (80.4)
Postgraduate degree	17 (4.1)
Monthly Family Income in Saudi Riyals (USD) (*n* = 392)	
5000 or less (1331 or less)	35 (8.9)
5001–7000 (1332–1864)	69 (17.6)
7001–10,000 (1865–2663)	64 (16.3)
10,001–15,000 (2664–3994)	74 (18.9)
15,001–20,000 (3995–5326)	56 (14.3)
Over 20,000 (over 5326)	94 (24.0)
Size of Home (*n* = 409)	
Traditional (folk)	4 (1.0)
Apartment	93 (22.7)
Small villa (<500 m^2^)	95 (23.2)
Medium (500–1000 m^2^)	175 (42.8)
Large (over 1000 m^2^)	42 (10.3)
Home Ownership (*n* = 413)	
Rented	109 (26.4)
Owned	297 (71.9)
Employer-provided	7 (1.7)
Number of Work Days/Week (*n* = 419)	
5	412 (98.1)
6	7 (1.7)
Number of Work Hours/Day (*n* = 412)	
1–4 h	15 (3.6)
5–6 h	134 (32.4)
7–8 h	197 (47.7)
9–10 h	60 (14.5)
Over 10 h	6 (1.5)
Job Description (*n* = 399)	
Supervisor	121 (28.8)
Non-supervisor	278 (66.2)
Physical Activity Social Support Score (*n* = 404)	
Mean (SD): 10.7 (±8.7)	
Low (<11)	194 (48.0)
High (≥11)	210 (52.0)
General Self-Efficacy Score (*n* = 417)	
Mean (SD): 3.05 (±0.5)	
Low (<3)	169 (41.0)
High (≥3)	248 (59.0)
BMI Category ^a^ (*n* = 393)	
Mean (SD): 27.1 (±5.9)	
Underweight	14 (3.6)
Normal	150 (38.2)
Overweight	127 (32.3)
Obese	102 (26.0)
PA level ^b^ (*n* = 420)	
Low	219 (52.1)
Moderate	173 (41.2)
High	28 (6.7)

^a^ Underweight: <18.5; Normal: 18.50–24.99, Overweight: 25.00–29.99, Obese: ≥30.0; ^b^ Low: <600 MET min/week, Moderate: 600–2999 MET min/week, High ≥1500 MET min/week vigorous activity or ≥3000 MET min/week moderate/vigorous PA; SD: standard deviation; USD: US dollar, BMI: body mass index; PA: physical activity.

**Table 2:** Self-reported sitting time (min/day) during various activities on work days and non-work days (*n* = 420).

Activity	Statistics (min/day)	Sitting Time (min/day)		
		Work Days	Non-Work Days	*p*-Value ^a^
Commuting	Mean (SD)	68.3 (53.1)	78.5 (73.1)	0.028
	Median (IQR)	60 (30–90)	60 (30–120)	
At work	Mean (SD)	323.3 (128.0)	N/A	N/A
	Median (IQR)	343.9 (240–420)		
Using home computer	Mean (SD)	67.4 (78.5)	99.7 (108.9)	<0.001
	Median (IQR)	60 (0–120)	60 (0–177)	
Watching TV	Mean (SD)	94.9 (84.9)	133.1 (116.6)	<0.001
	Median (IQR)	60 (30–120)	120 (55–180)	
At leisure ᵇ	Mean (SD)	126.7 (107.7)	255.0 (176.7)	<0.001
	Median (IQR)	120 (60–180)	240 (120–360)	
Total	Mean (SD)	680.9 (218.7)	565.8 (270.9)	<0.001
	Median (IQR)	690 (541–870)	575 (360–780)	

^a^ Wilcoxon Signed-Rank Test; ᵇ For example, socializing with friends or family, or talking on the phone; N/A: Not applicable; IQR: interquartile range.

**Table 3:** Beliefs about the effect of sedentary behavior on health.

Item	*n* (Valid Percent)					
	Strongly Agree	Agree	Don’t Know	Disagree	Strongly Disagree	*p*-Value
Sitting for prolonged periods can negatively impact my health (*n* = 418)	234 (56.0)	164 (39.2)	13 (3.1)	1 (0.2)	6 (1.4)	<0.001
Sitting for prolonged periods will not negatively impact my health if I regularly exercise after work (*n* = 410)	40 (9.8)	161 (39.3)	96 (23.4)	85 (20.7)	28 (6.8)	<0.001

**Table 4:** Median sitting time on work days and non-work days (min/day) for selected demographics, BMI, and physical activity levels of Saudi female employees.

Variables	Work Day Sitting (min/day) Median (IQR)	*p*-Value	Non-Work Day Sitting (min/day) Median (IQR)	*p*-Value
Age Range		0.001		<0.001
18−25	795 (630−960)		690 (555−960)	
26−35	720 (570−877)		625 (397−780)	
36−45	610 (467−737)		480 (307−585)	
46−60	630 (517−795)		540 (380−735)	
Marital Status		0.002		0.05
Single	780 (630−930)		660 (390−960)	
Married	665 (551−821)		370 (360−720)	
Divorced	682 (499−840)		545 (360−720)	
Widowed	767 (505−930)		690 (340−850)	
Number of Children		<0.001		0.001
0	780 (630−955)		660 (420−870)	
1	690 (572−855)		600 (450−720)	
2	645 (472−791)		630 (510−780)	
3	577.5 (476−709)		480 (322−700)	
4	602.5 (430−825)		480 (335−720)	
5 or more	480 (615−780)		432 (300−660)	
Education Level		<0.001		0.251
Primary school or lower	454 (328−682)		635 (267−960)	
Middle school	553 (350−757)		765 (367.5−960)	
High school	580 (465−720)		505 (346−742)	
College diploma/bachelor’s	720 (580−885)		600 (390−780)	
Postgraduate degree	770 (580−795)		600(500−750)	
Monthly Family Income in Saudi Riyal (USD)		0.028		0.945
5000 or less (1331 or less)	630 (385−777)		570 (380−960)	
5001−7000 (1332−1864)	720 (580−907)		630 (360−840)	
7001-10,000 (1865−2663)	765 (615−877)		630 (390−750)	
10,001−15,000 (2664−3994)	720 (585−870)		630 (390−832)	
15,001−20,000 (3995−5326)	667 (496−832)		575 (390−720)	
Over 20,000 (over 5326)	667 (555−890)		575 (390−720)	
Size of Home		0.561		0.139
Traditional (folk)	735 (585−772.5)		960 (300−960)	
Apartment	670 (530−825)		640 (475.5−750)	
Small villa (<500 m^2^)	695 (543.8–870)		532.5 (360−780)	
Medium (500−1000 m^2^)	710 (570−915)		630 (390−827.5)	
Large (over 1000 m^2^)	700 (592.5−865)		510 (322.5−780)	
Home Ownership		0.928		0.946
Rented	690 (551−870)		630 (390–735)	
Owned	700 (555−870)		600 (366−724)	
Employer-provided	780 (465−817)		540 (330−870)	
Number of Work Days/Week		0.508		0.286
5	700 (555−870)		600 (390−780)	
6	960 (365−960)		840 (360−960)	
Number of Work Hours/Day		0.039		0.212
1−4 h	600 (495−885)		600 (360−815)	
5−6 h	660 (499−840)		600 (387−750)	
7−8 h	710 (570−891.2)		540 (360−780)	
9−10 h	787.5 (620−930)		660 (436−840)	
Over 10 h	720 (540−885)		630 (540−960)	
Job Description		0.028		0.564
Supervisor	720 (573−900)		600 (362–780)	
Non-supervisor	660 (500−840)		570 (390−750)	
Physical Activity Social Support score		0.651		0.833
Low (<11)	690 (560–840)		600 (420–750)	
High (≥11)	712 (551–870)		600 (360–840)	
General Self-Efficacy score		0.862		0.293
Low (<3)	705 (555–870)		600 (390–780)	
High (≥3)	690 (540–845)		540 (355–757)	
BMI Category ^a^		0.008		0.121
Underweight	825 (690−960)		750 (611−870)	
Normal	720 (577−885)		600 (300−840)	
Overweight	705 (557−840)		550 (390−780)	
Obese	615 (480−825)		555 (360−780)	
PA level ^b^		0.140		0.171
Low	720 (570−872)		600 (360−780)	
Moderate	690 (540−870)		580 (390–750)	
High	660 (450−780)		720 (540−825)	

^a^ Underweight: <18.5, Normal: 18.50–24.99, Overweight: 25.00–29.99, Obese: ≥30.0; ^b^ Low: <600 MET min/week, Moderate: 600–2999 MET min/week, High ≥1500 MET min/week vigorous activity or ≥3000 MET min/week moderate/vigorous PA.

**Table 5:** Factors influencing work day and non-work day sitting time.

**Variable**	**Work Day Sitting Time**	
	**B (SE)**	**95% CI**
Constant	509.80 (76.35)	
Number of Children	−37.23 (6.18)	−49.38 to −25.07
Private Sector	79.75 (23.44)	33.62 to 125.88
Education Level	54.70 (19.03)	17.26 to 92.14
R^2^ (% of total variance explained)	0.21	
	**Non-Work Day Sitting Time**	
	**B (SE)**	**95% CI**
Constant	629.30 (23.96)	
Number of Children	−27.25 (8.80)	−44.56 to −9.95
Small House (ref. medium house)	−81.04 (32.88)	−145.74 to −16.35
Single (ref. married)	72.81 (36.10)	1.78 to 143.85
R^2^ (% of total variance explained)	0.09	
